# Association between depressive symptom and respiratory health in two prospective cohort studies

**DOI:** 10.1038/s41533-025-00473-3

**Published:** 2025-12-26

**Authors:** Xingjun Chen, Junyu Chen, Shuntao Lin, Hui Chen, Ziting Zhang, Li Wen, Xiaoxi Lu, Guangyan Liu

**Affiliations:** Affiliated Hospital of Guangdong Medical University, Zhanjiang, 524001 Guangdong China

**Keywords:** Diseases, Health care, Medical research, Risk factors

## Abstract

The association between depressive symptoms and respiratory health remains inconclusive, with limited research exploring dynamic changes in overall and symptom-specific depression. This study aimed to investigate the relationship between depressive symptom trajectories and the risk of chronic lung diseases (CLDs) as well as pulmonary function. We used data from two prospective cohorts: the China Health and Retirement Longitudinal Study (CHARLS) and the Health and Retirement Study (HRS). Depressive symptoms were assessed using the 10-item and 8-item CES-D scales, respectively, at three time points (CHARLS: wave1-3; HRS: wave 5–7), and classified into five trajectories: consistently low, decreasing, fluctuating, increasing, and consistently high. Incident CLDs were identified by self-reported physician diagnoses (CHARLS: wave 4–5; HRS: wave 8–12), and pulmonary function was evaluated by peak expiratory flow (PEF, CHARLS: wave 3; HRS: wave 8). Cox proportional hazards and linear regression models were used to estimate hazard ratios (HRs), beta coefficients (β), and 95% confidence intervals (CIs), adjusting for potential confounders. At baseline, individuals with depressive symptoms had a higher risk of Incident CLDs and lower PEF values. Compared to the consistently low group, the fluctuating (CHARLS: HR = 1.56, 95% CI: 1.33, 1.84; HRS: HR = 1.52, 95% CI: 1.30, 1.77), increasing (CHARLS: HR = 2.39, 95% CI: 1.86, 3.07; HRS: HR = 1.62, 95% CI: 1.13, 2.31), and consistently high (CHARLS: HR = 2.59, 95% CI: 2.16, 3.11; HRS: HR = 1.66, 95% CI: 1.30, 2.13) trajectories were associated with significantly increased CLDs risk. These trajectories were also significantly associated with lower PEF. The decreasing trajectory showed no significant association with CLDs risk or PEF. Total and somatic depressive symptoms demonstrated stronger associations with adverse respiratory outcomes. Depressive symptom trajectories characterized by fluctuation, increase, or persistent elevation are associated with higher CLDs risk and poorer pulmonary function. In contrast, symptom remission appears unrelated to respiratory outcomes. Total and somatic symptoms may serve as more sensitive indicators for predicting respiratory health.

## Introduction

Chronic lung diseases (CLDs), primarily comprising chronic obstructive pulmonary disease (COPD), represent a group of airways and pulmonary structural disorders. As one of the leading causes of disability and death worldwide, CLDs were responsible for approximately 3.91 million deaths in 2017, accounting for 7% of all-cause mortality globally^1^. In the same year, the global number of individuals living with CLDs reached nearly 545 million. With global population aging and increasing life expectancy, both the prevalence and absolute mortality of CLDs are expected to continue rising, posing a significant long-term challenge to healthcare systems worldwide^2^.

Beyond traditional risk factors such as smoking, environmental particulates, and air pollution, depressive symptoms represent an important psychosocial risk factor for CLDs^3,4^. Depressive symptoms comprise two dimensions: somatic symptoms and affective-cognitive symptoms^5^. Somatic symptoms include fatigue, sleep disturbances, changes in appetite, and psychomotor retardation, whereas affective-cognitive symptoms encompass depressed mood, loss of interest, and feelings of worthlessness^5^. Different dimensions of depressive symptoms have differential impacts on the risk of chronic disease. Compared with affective-cognitive symptoms, somatic depressive symptoms show stronger and more consistent associations with mortality and cardiovascular events among patients with heart disease^6–8^. Additionally, persistently high and fluctuating trajectories of somatic depressive symptoms increase the risk of developing diabetes, whereas trajectories of affective-cognitive symptoms do not appear to be associated with diabetes risk^9^. These differential associations between depressive symptom dimensions and chronic disease may be mediated by inflammatory and neuroendocrine responses^10,11^. Moreover, the longitudinal patterns of depressive symptoms exhibit substantial inter-individual heterogeneity. Some individuals experience only sporadic symptoms, whereas others endure persistently high levels of depressive symptom burden^12^. Persistent depressive symptoms may indicate the accumulation of more pronounced biological risk factors, making them stronger predictors of adverse health outcomes^13^.

Several observational studies have examined the relationship between depressive symptoms and CLDs. However, their findings have been inconsistent, and most studies used cross-sectional designs, which limit the ability to infer causality^14^. A longitudinal cohort study in China identified a bidirectional association between depressive symptoms and CLDs^3^, which is consistent with findings from a meta-analysis^15^. Nevertheless, these studies assessed depressive symptoms at only a single time point and did not account for their longitudinal changes. Compared with single-time-point assessments, evaluating depressive symptom trajectories over time may provide a more comprehensive understanding of their biological relevance. A recent study conducted in a European population identified four common depressive symptom trajectories, namely decreasing, fluctuating, increasing, and persistently high, all of which were significantly associated with incident CLDs risks^16^. However, the generalizability and robustness of these findings remain uncertain^16^. Furthermore, the study reported that improvement in affective-cognitive symptoms did not significantly reduce the risk of CLDs^16^, which challenges the effectiveness of cognitive-behavioral interventions in this patient population. Therefore, it is necessary to further explore the associations between depressive symptom trajectories, including their total and dimension-specific patterns, and respiratory health outcomes in diverse populations.

In the present study, we used two prospective cohort of China Health and Retirement Longitudinal Study (CHARLS) and Health and Retirement Study (HRS). We aimed to investigate the associations between trajectories of total, somatic, and affective-cognitive depressive symptoms and CLDs risks and lung function outcomes.

## Methods

### Study design and population

The CHARLS and HRS were all prospective and nationally representative cohorts conducted in China and the USA, respectively^17,18^. The CHARLS and HRS were approved by the Ethics Review Committees of Peking University, and the University of Michigan, respectively. Written informed consent was obtained from all participants. Detailed study designs on these three cohorts are summarized in *Supplementary Methods*. Wave 1(2011) of CHARLS and Wave 5(2000) of HRS were regarded as the baseline. Depressive symptom trajectories were assessed from baseline to the third wave in CHARLS (2015) and the seventh wave in HRS (2004). Lung function was evaluated at wave 3(2015) of CHARLS and Wave 8(2006) of HRS. Subsequent follow-up surveys were used to track the onset of CLDs until the final follow-up survey, which was Wave 5 (2020) of CHARLS and Wave 12 (2014) of HRS. The study design and strictly adhered to the STROBE Statement.

**Fig. 1:**
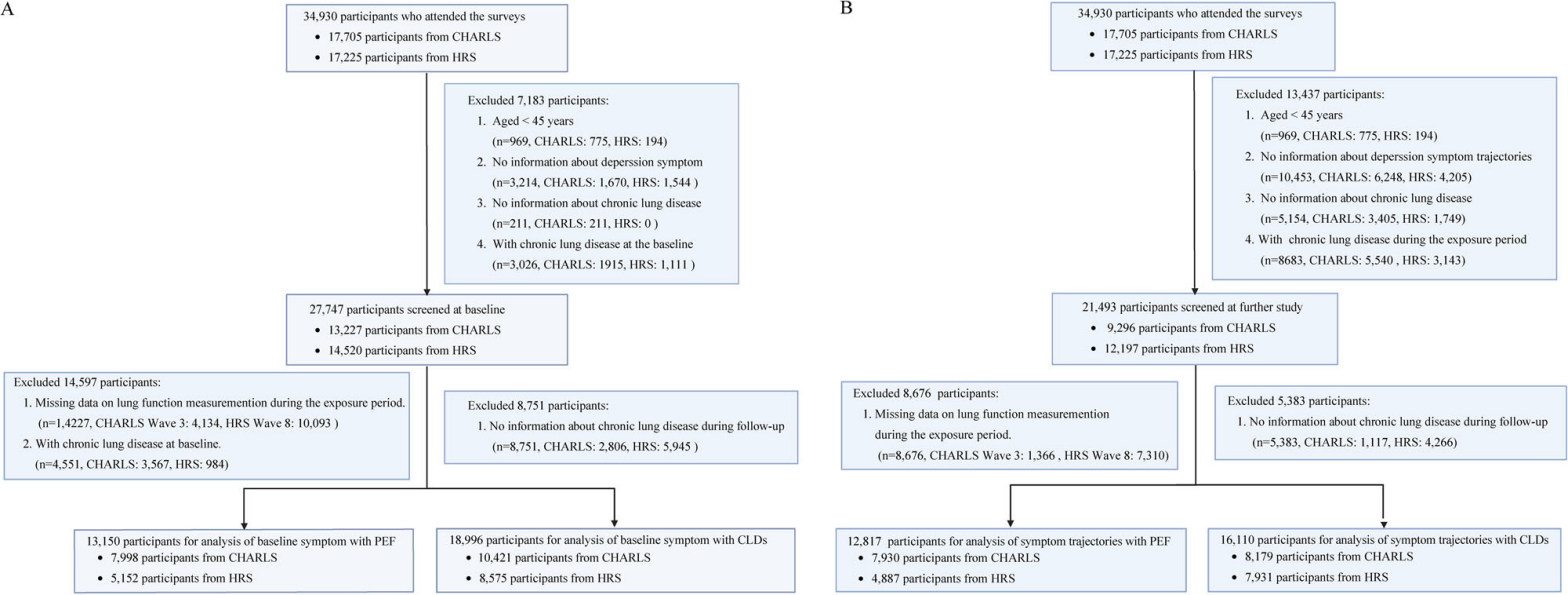
Selection process of the study population. **A** Analysis for baseline depressive symptoms with CLDs and PEF; **B** Analysis for depressive symptom trajectories with CLDs and PEF. CHARLS China Health and Retirement Longitudinal Study, HRS Health and Retirement Study, CLDs Chronic lung diseases, PEF Peak expiratory flow.

Figure 1 illustrates the selection process of the study population. At baseline, we excluded participants aged <45 years (n = 969), those without depressive symptom data (n = 3214), missing CLDs status (n = 211), or with existing CLDs (n = 3026). Among the remaining 27,747 participants, 8751 were lost to follow-up, leaving 18,996 for the baseline depressive symptoms and CLDs analysis. For the analysis of PEF as an outcome, we further excluded participants with CLDs during the exposure period (n = 4551) and those missing lung function data (n = 14,227), resulting in 13,150 participants. For the trajectory analysis, we excluded participants aged <45 years (n = 969), those without depressive symptom trajectories (n = 10,453), missing CLDs status (n = 5154), or with CLDs during the exposure period (n = 8683). After excluding 5383 lost to follow-up, 16,110 participants remained for the trajectory and CLDs analysis. For the PEF analysis related to trajectories, 8676 without lung function data during the exposure period were excluded, leaving 12,817 participants.

### Assessment of depressive symptoms

Depressive symptoms were assessed using the revised 10-item and 8-item versions of the Center for Epidemiologic Studies Depression Scale (CESD) in CHARLS and HRS, respectively. These scales have been validated in middle-aged and older adults, demonstrating good reliability, validity, and cross-cultural applicability^19,20^. In HRS, the CESD-8 includes eight binary (yes = 1 / no = 0) items referring to the presence of depressive symptoms during the past week, yielding a total score ranging from 0 to 8. In CHARLS, the CESD-10 assesses the frequency of ten depressive symptoms over the past week using a 4-point Likert scale, with a total score ranging from 0–30. The specific items of CESD-8 and CESD-10 are listed in Table S1. Consistent with previous studies, a CESD-8 score ≥3 or a CESD-10 score ≥10 was used to define significant overall depressive symptoms^21,22^. Based on a two-factor model, depressive symptoms were further categorized into cognitive-affective and somatic dimensions. The cognitive-affective dimension includes core negative emotions (e.g., ‘Feeling depressed’, ‘Feeling lonely’, or ‘Feeling sad/afraid’), while the somatic dimension includes physical symptoms (*e.g*., ‘Everything was an effort’, ‘Restless sleep’, and ‘Could not get going’)^23,24^. High symptom burden in each dimension was defined as scores in the top tertile: CESD-8 ≥ 2 or CESD-10 ≥ 6^25^.

### Depressive symptom trajectories

Five distinct depressive symptom trajectories (consistently low, decreasing, fluctuating, increasing, and consistently high) were predefined based on total CESD scores from CHARLS waves 1 to 3 and HRS waves 5–7. The consistently low trajectory was defined as no elevation in depressive symptoms across all three waves during the exposure period, whereas the consistently high trajectory was defined as elevated symptoms in all three waves. The decreasing trajectory was defined as (1) elevated baseline symptoms followed by reductions in the subsequent two waves; or (2) elevated symptoms in the first two waves but reduction in the third. The increasing trajectory was defined as (1) no elevated baseline symptoms with continuous increases in the following two waves; or (2) no elevation in the first two waves but elevation in the third. The fluctuating trajectory included all other depressive symptom patterns not meeting the above criteria (Table S2). The same definitions were applied to cognitive-emotional and somatic symptom trajectories.

### Assessment of CLDs and lung function

The primary outcomes of this study were incident CLDs and lung function. In CHARLS and HRS, CLDs were defined based on self-reported physician diagnoses, including chronic bronchitis, emphysema, and other pulmonary diseases, excluding asthma and lung cancer. Follow-up for baseline depressive symptoms and trajectory analyses began at wave 3 in CHARLS and wave 8 in HRS. The follow-up endpoint was the first occurrence of CLDs or censoring date. Peak expiratory flow (PEF) was used to assess lung function. PEF measurements were performed by trained technicians using a peak flow meter. During measurement, participants stood upright, took a deep breath, and blew into the peak flow meter as forcefully and quickly as possible, repeating the procedure three times. The maximum PEF value from wave 3 in CHARLS and wave 8 in HRS was used for analysis.

### Covariates

To ensure consistency between CHARLS and HRS, covariates in this study included age, sex, marital status, education, smoking status, drinking status, body mass index (BMI), hearing status, vision status, and medical history (hypertension, diabetes, heart disease, and stroke). Marital status was categorized into two groups: married or partnered, and other statuses (separated, divorced, single, or widowed). Education was classified into two levels: below high school, and high school or above. Smoking status was divided into never smokers and former or current smokers. Similarly, drinking status was categorized as never drinkers and drinkers. Hearing and vision status were classified as good or impaired. Details of the rationale for covariate selection were described in Supplementary Method.

### Statistical analysis

Baseline characteristics were summarized according to depressive symptom trajectories. Continuous variables were presented as means [standard deviations (SD)], whereas categorical variables were expressed as counts (percentages). Cox proportional hazards regression models were used to examine the associations of baseline depressive symptoms and depressive symptom trajectories with incident CLDs. Two Cox models were fitted using participants without depressive symptoms and those in the consistently low depressive symptom trajectory as references. Model 1 was unadjusted. Model 2 was adjusted for age, sex, marital status, education, smoking status, drinking status, BMI, hearing, vision, and medical history (hypertension, diabetes, heart disease, and stroke). The proportional hazards assumption was assessed using Kaplan-Meier curves, which appeared approximately parallel, and Schoenfeld residuals tests (all *P* > 0.05) (Figures S1 and S2, Supplementary Method). Additionally, linear regression analyses were performed to assess the associations between baseline depressive symptoms, depressive symptom trajectories, and lung function. Two models were fitted to estimate β coefficients and its 95% confidence intervals (95% CI). Residuals of the linear regression models met the assumptions of normality and homoscedasticity. The missing rates of covariates were summarized in Tables S3 and S4*.* Missing data for covariates were handled using chained-equation multiple imputation via the R package ‘mice’. We conducted five rounds of imputation to generate five datasets, setting the maximum number of iterations to 50 for each^26^.

Several sensitivity analyses were conducted: (i) Due to multiple comparisons, the primary analysis results were adjusted using the false discovery rate (FDR) correction. (ii) Given the ongoing debate regarding the cutoff for clinically significant depressive symptoms, higher thresholds were applied. Significant overall depressive symptoms were defined as CESD-10 ≥ 15 (CHARLS) or CESD-8 ≥ 4 (HRS). For cognitive-emotional and somatic symptoms, the thresholds were CESD-10 ≥ 9 (CHARLS) or CESD-8 ≥ 3 (HRS). (iii) Considering the competing risk between mortality and CLDs, we repeated the primary analyses using competing risks models. Finally, stratified analyses were conducted by sex, age (<65 years and ≥65 years), BMI (<24 kg/m² and ≥24 kg/m²), and smoking status. The statistical significance of interactions was assessed using likelihood ratio tests.

All statistical analyses were performed using *R* software (version 4.4.3). A two-sided *P* < 0.05 was considered statistically significant.

### Ethical approval

The China Health and Retirement Longitudinal Study was approved by the Ethics Review Committee of Peking University. The Health and Retirement Study was approved by the Ethics Review Committee of the University of Michigan. Informed consent was obtained from each subject in these two cohorts.

## Results

### Characteristics of participants

A total of 18,996 participants were included in the analysis of baseline depressive symptoms and CLDs, including 10,421 from CHARLS and 8575 from HRS. Additionally, 13,150 participants (CHARLS: 7998; HRS: 5152) were included in the baseline analysis of PEF. Baseline characteristics of these participants are presented in Table S5 and Table S6. For the analysis of depressive symptom trajectories and CLDs, 8179 participants from CHARLS (55.6% women; mean age: 57.6 years) and 7931 from HRS (64.5% women; mean age: 62.6 years) were included. In both CHARLS and HRS, participants in the persistently high symptom group were more likely to be female, married or partnered, consume alcohol, and have comorbid heart disease or stroke compared to those in the persistently low group (Table 1). In addition, based on the corresponding criteria, 7930 participants from CHARLS (55.2% women; mean age: 58.4 years) and 4887 from HRS (61.7% women; mean age: 69.2 years) were included in the trajectory and PFE analysis (Table 2). Baseline characteristics of participants included in the analysis and those excluded are presented in Table S7 and S8.

**Table 1:** Baseline characteristics of participants in the association analysis of depressive symptom trajectories and CLDs.

	CHARLS					HRS				
	Consistently low	Decreasing	Fluctuating	Increasing	Consistently high	Consistently low	Decreasing	Fluctuating	Increasing	Consistently high
**Participants, n (%)**	3676	396	2601	442	1064	5302	248	1725	205	451
**Age, mean (SD) years**	57 (8.0)	58 (9.0)	58 (8.0)	57 (8.0)	58 (8.0)	63 (8.0)	63 (8.0)	63 (8.0)	62 (8.0)	62 (8.0)
**Gender, n (%)**										
Female	1677 (45.6)	237 (59.8)	1561 (60.0)	291 (65.8)	784 (73.7)	3183 (60.0)	179 (72.2)	1246 (72.2)	161 (78.5)	353 (78.3)
Male	1999 (54.4)	159 (40.2)	1040 (40.0)	151 (34.2)	280 (26.3)	2119 (40.0)	69 (27.8)	479 (27.8)	44 (21.5)	98 (21.7)
**BMI, mean (SD), kg/m**^**2**^	24.6 (3.8)	23.6 (3.7)	24.0 (3.7)	24.0 (4.1)	23.5 (4.1)	29.0 (5.9)	29.7 (5.6)	29.5 (6.0)	29.0 (5.8)	30.0 (5.9)
**Marital status, n (%)**										
Married or partnered	3420 (93.0)	351 (88.6)	2317 (89.1)	393 (88.9)	912 (85.7)	4165 (78.6)	151 (60.9)	1176 (68.2)	146 (71.2)	249 (55.2)
Other marital status	256 (7.0)	45 (11.4)	284 (10.9)	49 (11.1)	152 (14.3)	1137 (21.4)	97 (39.1)	549 (31.8)	59 (28.8)	202 (44.8)
**Education level, n (%)**										
Below high school	3089 (84.0)	363 (91.7)	2387 (91.8)	400 (90.5)	1017 (95.6)	706 (13.3)	58 (23.4)	417 (24.2)	60 (29.3)	189 (41.9)
High school or above	587 (16.0)	33 (8.3)	214 (8.2)	42 (9.5)	47 (4.4)	4596 (86.7)	190 (76.6)	1308 (75.8)	145 (70.7)	262 (58.1)
**Smoking status, n (%)**										
Ever smokers	2158 (58.7)	259 (65.4)	1712 (65.8)	310 (70.1)	788 (74.1)	2438 (46.0)	115 (46.4)	755 (43.8)	80 (39.0)	196 (43.5)
Never smokers	1518 (41.3)	137 (34.6)	889 (34.2)	132 (29.9)	276 (25.9)	2864 (54.0)	133 (53.6)	970 (56.2)	125 (61.0)	255 (56.5)
**Drinking status, n (%)**										
Ever drinkers	2113 (57.5)	253 (63.9)	1666 (64.1)	306 (69.2)	743 (69.8)	2275 (42.9)	136 (54.8)	943 (54.7)	102 (49.8)	301 (66.7)
Never drinkers	1563 (42.5)	143 (36.1)	935 (35.9)	136 (30.8)	321 (30.2)	3027 (57.1)	112 (45.2)	782 (45.3)	103 (50.2)	150 (33.3)
**Hearing, n (%)**										
Fair or poor	1646 (44.8)	238 (60.1)	1530 (58.8)	238 (53.8)	735 (69.1)	634 (12.0)	58 (23.4)	279 (16.2)	35 (17.1)	100 (22.2)
Good	2030 (55.2)	158 (39.9)	1071 (41.2)	204 (46.2)	329 (30.9)	4668 (88.0)	190 (76.6)	1446 (83.8)	170 (82.9)	351 (77.8)
**Vision, n (%)**										
Fair or poor	2142 (58.3)	309 (78.0)	1895 (72.9)	282 (63.8)	863 (81.1)	558 (10.5)	70 (28.2)	354 (20.5)	40 (19.5)	167 (37.0)
Good	1534 (41.7)	87 (22.0)	706 (27.1)	160 (36.2)	201 (18.9)	4744 (89.5)	178 (71.8)	1371 (79.5)	165 (80.5)	284 (63.0)
**Hypertension, n (%)**										
No	2865 (77.9)	304 (76.8)	1942 (74.7)	327 (74.0)	785 (73.8)	3347 (63.1)	151 (60.9)	1000 (58.0)	110 (53.7)	225 (49.9)
Yes	811 (22.1)	92 (23.2)	659 (25.3)	115 (26.0)	279 (26.2)	1955 (36.9)	97 (39.1)	725 (42.0)	95 (46.3)	226 (50.1)
**Diabetes, n (%)**										
No	177 (4.8)	25 (6.3)	157 (6.0)	33 (7.5)	64 (6.0)	4887 (92.2)	213 (85.9)	1505 (87.2)	175 (85.4)	380 (84.3)
Yes	3499 (95.2)	371 (93.7)	2444 (94.0)	409 (92.5)	1000 (94.0)	415 (7.8)	35 (14.1)	220 (12.8)	30 (14.6)	71 (15.7)
**Heart disease, n (%)**										
No	3393 (92.3)	359 (90.7)	2345 (90.2)	392 (88.7)	892 (83.8)	4759 (89.8)	208 (83.9)	1489 (86.3)	180 (87.8)	379 (84.0)
Yes	283 (7.7)	37 (9.3)	256 (9.8)	50 (11.3)	172 (16.2)	543 (10.2)	40 (16.1)	236 (13.7)	25 (12.2)	72 (16.0)
**Stroke, n (%)**										
No	3629 (98.7)	386 (97.5)	2539 (97.6)	436 (98.6)	1034 (97.2)	5175 (97.6)	240 (96.8)	1664 (96.5)	199 (97.1)	424 (94.0)
Yes	47 (1.3)	10 (2.5)	62 (2.4)	6 (1.4)	30 (2.8)	127 (2.4)	8 (3.2)	61 (3.5)	6 (2.9)	27 (6.0)

*CLDs* Chronic lung diseases, *CHARLS* China Health and Retirement Longitudinal Study, *HRS* Health and Retirement Study, *BMI* body mass index.

**Table 2:** Baseline characteristics of participants in the association analysis of depressive symptom trajectories and PEF.

	CHARLS					HRS				
	Consistently low	Decreasing	Fluctuating	Increasing	Consistently high	Consistently low	Decreasing	Fluctuating	Increasing	Consistently high
**Participants, n**	3537	384	2555	421	1033	3204	147	1192	54	290
**Age, mean (SD), years**	58 (9.0)	60 (9.0)	58 (9.0)	58 (8.0)	58 (8.0)	64 (9.0)	65 (9.0)	65 (9.0)	65 (10.0)	65 (10.0)
**Gender, n (%)**										
Female	1588 (44.9)	224 (58.3)	1525 (59.7)	286 (67.9)	757 (73.3)	1832 (57.2)	100 (68.0)	830 (69.6)	40 (74.1)	212 (73.1)
Male	1949 (55.1)	160 (41.7)	1030 (40.3)	135 (32.1)	276 (26.7)	1372 (42.8)	47 (32.0)	362 (30.4)	14 (25.9)	78 (26.9)
**BMI, mean (SD), kg/m2**	24.6 (3.8)	23.5 (3.7)	24.0 (4.7)	23.9 (4.3)	23.2 (3.8)	28 (6.0)	28 (7.0)	29 (6.0)	28 (7.0)	30 (7.0)
**Marital status, n (%)**										
Married or partnered	3274 (92.6)	326 (84.9)	2256 (88.3)	370 (87.9)	881 (85.3)	2528 (78.9)	96 (65.3)	842 (70.6)	35 (64.8)	167 (57.6)
Other marital status	263 (7.4)	58 (15.1)	299 (11.7)	51 (12.1)	152 (14.7)	676 (21.1)	51 (34.7)	350 (29.4)	19 (35.2)	123 (42.4)
**Education level, n (%)**										
Below high school	3004 (84.9)	358 (93.2)	2352 (92.1)	391 (92.9)	990 (95.8)	438 (13.7)	35 (23.8)	294 (24.7)	22 (40.7)	118 (40.7)
High school or above	533 (15.1)	26 (6.8)	203 (7.9)	30 (7.1)	43 (4.2)	2766 (86.3)	112 (76.2)	898 (75.3)	32 (59.3)	172 (59.3)
**Smoking status, n (%)**										
Ever smokers	1488 (42.1)	140 (36.5)	882 (34.5)	133 (31.6)	285 (27.6)	1743 (54.4)	86 (58.5)	654 (54.9)	31 (57.4)	164 (56.6)
Never smokers	2049 (57.9)	244 (63.5)	1673 (65.5)	288 (68.4)	748 (72.4)	1461 (45.6)	61 (41.5)	538 (45.1)	23 (42.6)	126 (43.4)
**Drinking status, n (%)**										
Ever drinkers	1517 (42.9)	140 (36.5)	908 (35.5)	126 (29.9)	316 (30.6)	1778 (55.5)	67 (45.6)	556 (46.6)	22 (40.7)	106 (36.6)
Never drinkers	2020 (57.1)	244 (63.5)	1647 (64.5)	295 (70.1)	717 (69.4)	1426 (44.5)	80 (54.4)	636 (53.4)	32 (59.3)	184 (63.4)
**Hearing, n (%)**										
Fair or poor	1634 (46.2)	232 (60.4)	1504 (58.9)	231 (54.9)	708 (68.5)	458 (14.3)	29 (19.7)	193 (16.2)	16 (29.6)	65 (22.4)
Good	1903 (53.8)	152 (39.6)	1051 (41.1)	190 (45.1)	325 (31.5)	2746 (85.7)	118 (80.3)	999 (83.8)	38 (70.4)	225 (77.6)
**Vision, n (%)**										
Fair or poor	2146 (60.7)	304 (79.2)	1848 (72.3)	282 (67.0)	838 (81.1)	368 (11.5)	31 (21.1)	256 (21.5)	20 (37.0)	106 (36.6)
Good	1391 (39.3)	80 (20.8)	707 (27.7)	139 (33.0)	195 (18.9)	2836 (88.5)	116 (78.9)	936 (78.5)	34 (63.0)	184 (63.4)
**Hypertension, n (%)**										
No	2720 (76.9)	296 (77.1)	1894 (74.1)	312 (74.1)	743 (71.9)	1947 (60.8)	84 (57.1)	663 (55.6)	28 (51.9)	141 (48.6)
Yes	817 (23.1)	88 (22.9)	661 (25.9)	109 (25.9)	290 (28.1)	1257 (39.2)	63 (42.9)	529 (44.4)	26 (48.1)	149 (51.4)
**Diabetes, n (%)**										
No	3354 (94.8)	356 (92.7)	2398 (93.9)	390 (92.6)	963 (93.2)	2895 (90.4)	127 (86.4)	1017 (85.3)	43 (79.6)	241 (83.1)
Yes	183 (5.2)	28 (7.3)	157 (6.1)	31 (7.4)	70 (6.8)	309 (9.6)	20 (13.6)	175 (14.7)	11 (20.4)	49 (16.9)
**Heart disease, n (%)**										
No	3245 (91.7)	348 (90.6)	2287 (89.5)	371 (88.1)	854 (82.7)	2810 (87.7)	116 (78.9)	984 (82.6)	42 (77.8)	232 (80.0)
Yes	292 (8.3)	36 (9.4)	268 (10.5)	50 (11.9)	179 (17.3)	394 (12.3)	31 (21.1)	208 (17.4)	12 (22.2)	58 (20.0)
**Stroke, n (%)**										
No	3491 (98.7)	374 (97.4)	2495 (97.7)	415 (98.6)	1000 (96.8)	3100 (96.8)	141 (95.9)	1139 (95.6)	51 (94.4)	274 (94.5)
Yes	46 (1.3)	10 (2.6)	60 (2.3)	6 (1.4)	33 (3.2)	104 (3.2)	6 (4.1)	53 (4.4)	3 (5.6)	16 (5.5)
**PEF, mean (SD), L/min**	344 (123.0)	307 (120.0)	309 (113.0)	301 (113.0)	277 (106.0)	64 (9.0)	65 (9.0)	65 (9.0)	65 (10.0)	65 (10.0)

*PEF* Peak expiratory flow, *CLDs* Chronic lung diseases, *CHARLS* China Health and Retirement Longitudinal Study, *HRS* Health and Retirement Study, *BMI* body mass index.

In the baseline depressive symptom analysis, the median follow-up was 9.0 years in CHARLS and 14.0 years in HRS. A total of 464 participants (248 from CHARLS and 216 from HRS) died during follow-up. In the depressive symptom trajectory analysis, the median follow-up periods were 5.0 years in CHARLS and 9.9 years in HRS. 1341 participants (569 from CHARLS and 772 from HRS) died during follow-up.

### Association of baseline depressive symptoms with incident CLDs

Table 3 presents the associations between baseline depressive symptoms and the risk of incident CLDs. After adjusting for confounders, participants with depressive symptoms had a significantly higher risk of CLDs compared to those without symptoms (CHARLS: HR = 1.61, 95% CI: 1.46, 1.77; HRS: HR = 1.53, 95% CI: 1.35, 1.73). Similarly, participants with somatic or cognitive-emotional symptoms also exhibited a significantly increased risk of CLDs compared to healthy individuals. For somatic symptoms: CHARLS, HR = 1.52, 95% CI: 1.38, 1.66; HRS, HR = 1.61, 95% CI: 1.42–1.83. For cognitive-emotional symptoms: CHARLS, HR = 1.37, 95% CI: 1.24–1.51; HRS, HR = 1.42, 95% CI: 1.25–1.61.

**Table 3:** Association of baseline depression symptom with risks of incident CLDs.

	CHRALS					HRS				
	events/n	HR1 (95%CI)^1^	*P1*^1^	HR2 (95%CI)^2^	*P2*^2^	events/n	HR1 (95%CI)^1^	*P1*^1^	HR2 (95%CI)^2^	*P2*^2^
**Total depressive symptom**										
No	1002/6728	Ref		Ref		981/6919	Ref		Ref	
Yes	884/3693	1.69 (1.54, 1.84)	<0.001	1.61 (1.46, 1.77)	<0.001	406/1656	1.85 (1.65, 2.08)	<0.001	1.53 (1.35, 1.73)	<0.001
**Somatic depressive symptom**										
No	988/6496	Ref		Ref		1006/7087	Ref		Ref	
Yes	898/3925	1.58 (1.44, 1.73)	<0.001	1.52 (1.38, 1.66)	<0.001	381/1488	1.96 (1.74, 2.20)	<0.001	1.61 (1.42, 1.83)	<0.001
**Cognitive affective depressive symptom**										
No	965/6208	Ref		Ref		1030/7076	Ref		Ref	
Yes	921/4213	1.45 (1.33, 1.59)	<0.001	1.36 (1.24, 1.49)	<0.001	357/1499	1.73 (1.54, 1.95)	<0.001	1.42 (1.25, 1.61)	<0.001

*CHARLS* China Health and Retirement Longitudinal Study, *HRS* Health and Retirement Study, *CLDs* Chronic lung diseases.

^1^HR1 and P1 were unadjusted.

^2^HR2 and P2 were adjusted for age, gender, BMI, education level, marital status, smoking status, drinking status, hearing, vision, hypertension, diabetes, heart disease, and stroke.

### Association of baseline depressive symptoms with PEF

Table 4 presents the associations between baseline depressive symptoms and PEF. After adjusting for confounding factors, participants with depressive symptoms had a significantly higher risk of reduced PEF compared to healthy individuals (CHARLS: β = −15.66, 95% CI: -20.25, −11.06; HRS: β = −12.96, 95% CI: −19.93, −5.99). Similarly, participants exhibiting somatic symptoms (CHARLS: β = −10.61, 95% CI: −15.09, −6.13; HRS: β = −15.46, 95% CI: −22.76, −8.16) or cognitive-affective symptoms (CHARLS: β = −13.22, 95% CI: −17.64, −8.80; HRS: β = −12.84, 95% CI: −20.10, −5.58) also showed a significant association with lower PEF levels compared to healthy individuals.

**Table 4:** Association of baseline depression symptom with PEF.

	CHRALS					HRS				
	n	β1 (95%CI)^1^	*P1*^1^	β2 (95%CI)^2^	*P2*^2^	n	β1 (95%CI)^1^	*P1*^1^	β2 (95%CI)^2^	*P2*^2^
**Total depressive symptom**										
No	5204	Ref		Ref		4176	Ref		Ref	
Yes	2794	−44.08 (−50.42, −39.53)	<0.001	−15.66 (−20.25, −11.06)	<0.001	976	−39.70 (−48.55, −30.85)	<0.001	−12.96 (−19.93, −5.99)	<0.001
**Somatic depressive symptom**										
No	5000	Ref		Ref		4278	Ref		Ref	
Yes	2998	−38.38 (−43.76, −33.00)	<0.001	−10.61 (−15.09, −6.13)	<0.001	874	−39.70 (−48.95, −30.45)	<0.001	−15.46 (−22.76, −8.16)	<0.001
**Cognitive affective depressive symptom**										
No	3232	Ref		Ref		4293	Ref		Ref	
Yes	4766	−34.93 (−29.61, −40.25)	<0.001	−13.22 (−8.80, −17.64)	<0.001	859	−40.69 (−50.00, −31.38)	<0.001	−12.84 (−20.10, −5.58)	<0.001

*CHARLS* China Health and Retirement Longitudinal Study, *HRS* Health and Retirement Study, *PEF* Peak expiratory flow.

^1^β1 and P1 were unadjusted.

^2^β2 and P2 were adjusted for age, gender, BMI, education level, marital status, smoking status, drinking status, hearing, vision, hypertension, diabetes, heart disease, and stroke.

### Association of depressive symptoms trajectories with incident CLDs

Table 5 presents the associations between depressive symptom trajectories and incident CLDs risks. Participants with consistently high depressive symptoms had a significantly higher risk of incident CLDs compared to those with consistently low symptoms (CHARLS: HR = 2.59, 95% CI: 2.16, 3.11; HRS: HR = 1.66, 95% CI: 1.30, 2.13). Similarly, participants with fluctuating (CHARLS: HR = 1.56, 95% CI: 1.33, 1.84; HRS: HR = 1.52, 95% CI: 1.30, 1.77) or increasing depressive symptoms (CHARLS: HR = 2.39, 95% CI: 1.86, 3.07; HRS: HR = 1.62, 95% CI: 1.13, 2.31) also showed elevated risks compared to those with persistently low symptoms. However, the association was not statistically significant among participants with decreasing depressive symptoms (CHARLS: HR = 1.30, 95% CI: 0.94, 1.78; HRS: HR = 1.37, 95% CI: 0.97, 1.94).

**Table 5:** Cox proportional hazard ratios for the association of depressive symptom trajectories with CLDs.

	CHRALS (n = 8179)					HRS (n = 7931)				
	events/n	HR1 (95%CI)^1^	*P1*^1^	HR2 (95%CI)^2^	*P2*^2^	n	HR1 (95%CI)^1^	*P1*^1^	HR2 (95%CI)^2^	*P2*^2^
**Total depressive symptom trajectory**										
Consistently low	299/3676	Ref		Ref		478/5302	Ref		Ref	
Decreasing	44/396	1.38 (1.00, 1.89)	0.047	1.30 (0.94, 1.78)	0.11	35/248	1.60 (1.13, 2.25)	0.008	1.37 (0.97, 1.94)	0.751
Fluctuating	336/2601	1.62 (1.39, 1.90)	<0.001	1.56 (1.33, 1.84)	<0.001	261/1725	1.71 (1.47, 1.99)	<0.001	1.52 (1.30, 1.77)	<0.001
Increasing	79/442	2.36 (1.84, 3.02)	<0.001	2.39 (1.86, 3.07)	<0.001	33/205	1.85 (1.30, 2.64)	<0.001	1.62 (1.13, 2.31)	0.008
Consistently high	222/1064	2.71 (2.28, 3.22)	<0.001	2.59 (2.16, 3.11)	<0.001	83/451	2.09 (1.66, 2.64)	<0.001	1.66 (1.30, 2.13)	<0.001
**Somatic symptom trajectory**										
Consistently low	278/3285	Ref		Ref		494/5442	Ref		Ref	
Decreasing	16/175	1.04 (0.63, 1.72)	0.875	0.99 (0.60, 1.64)	0.974	12/90	1.51 (0.85, 2.68)	0.155	1.45 (0.82, 2.58)	0.205
Fluctuating	436/3482	1.46 (1.26, 1.69)	<0.001	1.40 (1.21, 1.64)	<0.001	301/1971	1.72 (1.49, 1.99)	<0.001	1.52 (1.30, 1.76)	<0.001
Increasing	65/380	2.07 (1.58, 2.70)	<0.001	2.04 (1.55, 2.67)	<0.001	9/40	2.54 (1.32, 4.92)	0.005	2.66 (1.37, 5.16)	0.004
Consistently high	176/857	2.48 (2.06, 2.99)	<0.001	2.35 (1.93, 2.86)	<0.001	74/388	2.18 (1.71, 2.79)	<0.001	1.74 (1.34, 2.25)	<0.001
**Cognitive affective symptom trajectory**										
Consistently low	175/2288	Ref		Ref		517/5439	Ref		Ref	
Decreasing	41/370	1.49 (1.06, 2.09)	0.022	1.34 (0.95, 1.89)	0.095	16/163	1.01 (0.61, 1.66)	0.980	0.91 (0.55, 1.50)	0.707
Fluctuating	469/3838	1.64 (1.38, 1.95)	<0.001	1.54 (1.29, 1.84)	<0.001	267/1819	1.58 (1.36, 1.83)	<0.001	1.41 (1.21, 1.64)	<0.001
Increasing	88/599	1.99 (1.54, 2.57)	<0.001	1.89 (1.46, 2.44)	<0.001	22/142	1.67 (1.09, 2.55)	0.019	1.55 (1.01, 2.38)	0.047
Consistently high	207/1084	2.63 (2.15, 3.21)	<0.001	2.38 (1.93, 2.93)	<0.001	68/368	1.94 (1.51, 2.50)	<0.001	1.51 (1.15, 1.96)	0.003

*CHARLS* China Health and Retirement Longitudinal Study, *HRS* Health and Retirement Study, *CLD* Chronic lung disease.

^1^HR1 and P1 were unadjusted.

^2^HR2 and P2 were adjusted for age, gender, BMI, education level, marital status, smoking status, drinking status, hearing, vision, hypertension, diabetes, heart disease, and stroke.

Similar patterns were observed for trajectories of somatic and cognitive-affective symptoms. Compared with the persistently low group, participants in the fluctuating, increasing, or persistently high trajectory groups had a significantly higher incident CLDs risks (*P* < 0.05). In contrast, decreasing somatic or cognitive-affective symptoms were not significantly associated with the risk of CLDs.

### Association of depressive symptoms trajectories with PEF

Table 6 presents the associations between depressive symptom trajectories and PEF. In CHARLS, compared with participants with persistently low depressive symptoms, those with fluctuating (β = −16.62, 95% CI: −21.45, −11.79), increasing (β = −14.07, 95% CI: −23.32, −4.82), and persistently high symptoms (β = −31.70, 95% CI: −38.17, −25.22) showed significantly lower PEF levels. Similar results were observed in HRS. Regarding somatic and cognitive-affective symptom trajectories, the fluctuating, increasing, and persistently high patterns were significantly associated with lower PEF in CHARLS and HRS compared to the persistently low trajectory group (*P* < 0.05). Additionally, in CHARLS and HRS, a decreasing trajectory in cognitive-affective symptoms was significantly associated with reduced PEF.

**Table 6:** Association of depressive symptom trajectories with PEF.

	CHRALS					HRS				
	n	β1 (95%CI)^1^	*P1*^1^	β2 (95%CI)^2^	*P2*^2^	n	β1 (95%CI)^1^	*P1*^1^	β2 (95%CI)^2^	*P2*^2^
**Total depressive symptom trajectory**										
Consistently low	3537	Ref		Ref		3204	Ref		Ref	
Decreasing	384	−37.23 (−49.55, −24.91)	<0.001	−7.87 (−17.73, 1.99)	0.118	147	−37.38 (−58.14, −16.63)	<0.001	−11.45 (−27.24, 4.35)	0.156
Fluctuating	2555	−35.44 (−41.39, −29.48)	<0.001	−16.62 (−21.45, −11.79)	<0.001	1192	−39.84 (−48.19, −31.49)	<0.001	−13.39 (−19.86, −6.92)	<0.001
Increasing	421	−43.07 (−54.89, −31.24)	<0.001	−14.07 (−23.32, −4.82)	0.003	54	−59.66 (−93.42, −25.90)	<0.001	−18.78 (−44.54, −6.98)	0.024
Consistently high	1033	−66.73 (−74.84, −58.62)	<0.001	−31.70 (−38.17, −25.22)	<0.001	290	−68.24 (−83.33, −53.16)	<0.001	−26.14 (−37.96, −14.32)	<0.001
**Somatic symptom trajectory**										
Consistently low	3146	Ref		Ref		3332	Ref		Ref	
Decreasing	185	−14.85 (−32.26, 2.57)	0.095	−4.68 (−18.85, 9.49)	0.517	87	−18.95 (−45.73, 7.83)	0.166	−2.33 (−22.64, 17.98)	0.822
Fluctuating	3399	−37.06 (−42.75, −31.36)	<0.001	−10.20 (−14.95, −5.46)	<0.001	1142	−38.09 (−46.54, −29.64)	<0.001	−16.25 (−22.82, −9.68)	<0.001
Increasing	368	−33.42 (−46.11, −20.74)	<0.001	−9.57 (−19.94, −1.82)	0.022	83	−57.49 (−84.89, −30.09)	<0.001	−18.08 (−31.73, −9.97)	0.036
Consistently high	832	−62.14 (−71.11, −53.16)	<0.001	−19.17 (−26.69, −11.64)	<0.001	243	−65.92 (−82.31, −49.54)	<0.001	−27.16 (−39.90, −14.42)	<0.001
**Cognitive affective symptom trajectory**										
Consistently low	2166	Ref		Ref		3337	Ref		Ref	
Decreasing	353	−56.51 (−69.72, −43.30)	<0.001	−21.69 (−32.51, −10.87)	<0.001	96	−46.26 (−71.79, −20.74)	<0.001	−19.66 (−39.07, −0.25)	0.047
Fluctuating	3766	−38.54 (−44.74, −32.33)	<0.001	−13.24 (−18.38, −8.11)	<0.001	1188	−35.04 (−43.37, −26.70)	<0.001	−10.51 (−16.93, −4.08)	0.001
Increasing	1059	−63.17 (−71.80, −54.54)	<0.001	−26.59 (−33.80, −19.38)	<0.001	40	−82.26 (−121.49, −43.04)	<0.001	−36.88 (−66.67, −7.08)	0.015
Consistently high	586	−45.78 (−56.49, −35.06)	<0.001	−17.00 (−25.75, −8.24)	<0.001	226	−67.62 (−84.57, −50.67)	<0.001	−22.74 (−35.93, −9.55)	<0.001

*CHARLS* China Health and Retirement Longitudinal Study, *HRS* Health and Retirement Study, *PEF* Peak expiratory flow.

^1^β1 and P1 were unadjusted.

^2^β2 and P2 were adjusted for age, gender, BMI, education level, marital status, smoking status, drinking status, hearing, vision, hypertension, diabetes, heart disease, and stroke.

### Sensitivity analyses

After FDR correction, the statistical significance of the primary analyses remained unchanged (Tables S9-S12). Similar results were revealed in both CHARLS and HRS when defining clinically significant depressive symptoms using a higher threshold (Tables S13-S16). Furthermore, the results were consistent with the main analyses after further conducting the competing risk analyses between mortality and CLDs (Tables S17 and S18). In stratified analyses, similar associations were observed among middle-aged and older adults, men and women, individuals with normal weight and obesity, and across different smoking statuses (Tables S19-S24).

## Discussion

In this study involving two prospective cohort datasets, we examined the associations of baseline depressive symptoms and depressive symptom trajectories with the risk of incident CLDs and PEF. Participants with overall depressive symptoms, somatic symptoms, or cognitive-affective symptoms consistently showed an elevated risk of incident CLDs and significantly lower PEF compared to those without such symptoms. Across total, somatic, and cognitive-affective symptom trajectories, participants with fluctuating, increasing, or persistently high symptoms had significantly higher CLDs risk compared to those with persistently low symptoms. Additionally, these trajectory groups were generally associated with reduced PEF. These findings remained robust across multiple sensitivity analyses. To our knowledge, this is the first study investigating the associations between depressive symptom trajectories, including overall, somatic, and cognitive-emotional subtypes, and CLDs risk as well as PEF levels among middle-aged and older adults in China and the United States.

Depression is a common and severe comorbidity among patients with COPD, significantly affecting the progression and prognosis of CLDs. A large meta-analysis reported that COPD patients with comorbid depression exhibited higher rates of acute exacerbations and hospital readmissions^27^. In COPD populations, depressive symptoms explained a greater proportion of variance in patient-reported outcomes (PROs) than pulmonary function measures such as forced expiratory volume (FEV%)^28^. However, few studies have investigated whether depressive symptoms increase the risk of developing CLDs and affect lung function in healthy populations across different countries. A cohort study in Korea found that individuals with depression had higher risks of respiratory symptoms and chronic bronchitis, as well as lower diffusing capacity for carbon monoxide (DLCO), compared to those without depression^29^. A Canadian national health survey reported that respondents with severe depression at baseline had a 2.1-fold increased risk of developing COPD compared to those without depression^30^. Studies based on Chinese populations also support that depressive symptoms predict a higher risk of CLDs. In a German community cohort of over 12,000 individuals, each additional point in depressive symptom score was associated with an 8% increase in CLDs incidence^31^. These findings are consistent with our baseline analyses. Moreover, our study revealed a significant association between depressive symptoms and declines in PEF, further highlighting the importance of addressing depressive symptoms in healthy populations. Considering the persistence and dynamic nature of depressive symptoms, we reconstructed symptom trajectories to rigorously examine their impact on respiratory health. Our results indicated that persistently high levels of depressive symptoms significantly increased the risk of incident CLDs and reduced PEF, which may be attributed to cumulative damage to the respiratory system over time. In contrast, individuals with decreasing depressive symptom trajectories did not show significant associations with incident CLDs or PEF. This finding suggests that individuals experiencing high levels of depressive symptoms may require sufficient recovery time to avoid increased risk of respiratory system impairment.

Somatic and cognitive-emotional symptoms represent two distinct dimensions of overall depressive symptoms. In U.S. veterans with COPD, both more severe overall depressive symptoms and somatic symptoms have been significantly associated with fewer daily steps^32^. Chronic somatic symptoms may also contribute to adverse lifestyle behaviors, including sleep disturbances, physical inactivity, and dependence on smoking^33–35^. These behaviors have been shown to increase incident CLDs and are directly linked to impaired pulmonary function^36^. Sustained physical inactivity, in particular, not only elevates the risk of acute exacerbations in COPD but is also positively associated with accelerated decline in lung function, such as reduced FEV1^37,38^. These mechanisms may help explain why overall and somatic depressive symptoms are stronger predictors of incident CLDs. However, in contrast to previous findings, our results demonstrate that cognitive-emotional depressive symptoms also contribute to incident CLDs and lung function impairment. These symptoms involve emotional, behavioral, and social withdrawal patterns. Depression-related cognitive impairments are often accompanied by reduced self-efficacy and poor self-care behaviors^39^, leading to higher likelihood of smoking dependence as a form of emotional coping^40^. Such behaviors may trigger systemic inflammation and increase vulnerability to CLDs^41^. Furthermore, prolonged negative mood, social isolation, and avoidance of social engagement may weaken patients’ ability to manage their respiratory health^42^. Therefore, psychological interventions, including cognitive behavioral therapy combined with exercise training, have been shown to more effectively improve depressive symptoms in individuals with CLDs, aligning with the conclusions of most prior studies^43–45^.

Biological pathways link depressive symptoms with CLDs. Depressive symptoms induce chronic stress that persistently activates the hypothalamic-pituitary-adrenal (HPA) axis, resulting in abnormal cortisol secretion. This activation promotes systemic inflammation, driving chronic airway inflammation and lung tissue damage^5^. Additionally, depressive symptoms provoke sympathetic nervous system overactivity and reduced vagal tone^46^. These autonomic disturbances stimulate catecholamine release, which in turn triggers the secretion of proinflammatory cytokines, exacerbating airway inflammation and airflow limitation^47^. Furthermore, serotonin, a monoamine neurotransmitter associated with depressive symptoms, suppresses cytokine production by type 2 innate lymphoid cells (ILC2), such as interleukin-5 and interleukin-13, thereby alleviating pulmonary inflammation^48^. Overall, systemic and local inflammation represent core mechanisms underlying the impact of depression on CLDs progression^41^. Somatic depressive symptoms elicit stronger inflammatory responses than cognitive-emotional symptoms^5^. Beyond these general mechanisms, trajectory-specific pathways may exist. For example, increasing depressive symptoms may accelerate the onset of comorbid conditions such as asthma and hysteria, further elevating CLDs risk^49^. Fluctuating depressive symptoms resemble weight variability, potentially causing greater inflammatory or biological pathway dysregulation^50^. Additional research is required to elucidate these trajectory-specific mechanisms.

The core strength of this study lies in the utilization of two nationally representative large cohorts from China and the United States, enabling dynamic characterization of depressive symptom trajectories through repeated longitudinal assessments. Furthermore, the study design substantially enhances the ability to discern complex associations between symptom patterns, incident CLDs, and PEF. The innovative dimensional analysis of depressive symptoms, distinguishing somatic and cognitive-emotional domains, reveals differential associations with CLDs risk and lung function, providing a theoretical basis for targeted interventions. In addition, the study population was drawn from general community samples, which ensures the robustness and generalizability of the findings. Nevertheless, several limitations warrant cautious interpretation. First, the study cohorts comprised middle-aged and older adults aged 45 years, which limits generalizability to younger populations and other geographic regions. Second, depressive symptoms were assessed using the CES-D screening tool rather than clinical diagnosis, and the symptoms themselves are inherently variable, the effect estimates are likely attenuated toward the null. The dimensional categorization of depressive symptoms was an exploratory attempt based on prior literature and has not been statistically validated. Third, this study did not utilize the full continuous CES-D score information to define depressive symptom trajectories. Future research should explore alternative modeling approaches, such as growth-based trajectory modeling (GBTM), to further validate and refine these findings. Fourth, although multiple covariates were adjusted for, residual confounding may still exist due to unmeasured factors such as occupational exposures, physical activity, socioeconomic conditions, and medication adherence related to CLDs. Fifth, due to the design of the CHARLS study, depressive symptom trajectories overlapped with the timing of PEF measurement, preventing lagged analyses. Sixth, compared to FEV1and FVC, PEF is not the optimal measure of pulmonary function. Finally, dichotomization of certain covariates to simplify the model may introduce residual confounding bias.

Based on the findings of this study, we recommend incorporating systematic screening and dynamic management of depressive symptoms, particularly their somatic dimensions, into routine prevention and clinical management strategies for chronic pulmonary diseases. This approach involves not only monitoring pulmonary function in respiratory and general outpatient settings but also routinely assessing patients’ psychological status. Special attention should be given to patients reporting long-term fatigue, sleep disturbances, appetite changes, and other somatic manifestations of depression. In clinical practice, comprehensive intervention plans should be developed for patients with comorbid depressive symptoms and chronic lung disease, integrating pharmacotherapy, psychotherapy, and pulmonary rehabilitation to simultaneously improve psychological well-being and physiological function. Furthermore, periodic reassessment of depressive symptom scores alongside pulmonary function is recommended, with treatment strategies dynamically adjusted based on this dual evaluation. Such an approach can effectively alleviate depressive symptoms, slow the decline of pulmonary function, and ultimately reduce the risk of disease onset and acute exacerbations in patients with chronic pulmonary disease.

## Conclusion

Baseline depressive symptoms, as well as fluctuating, increasing, and persistently high symptom trajectories, were significantly associated with elevated risk of incident CLDs and reduced lung function. In contrast, decreasing depressive symptom trajectories showed no significant association with CLDs risk or lung function. Stronger correlations were observed for overall and somatic depressive symptoms with both CLDs incidence and pulmonary function. This study highlights the clinical importance of monitoring depressive symptom trajectories and implementing targeted screening for somatic symptoms among middle-aged and older adults to identify individuals at high risk for incidents CLDs. Future research should further elucidate the specific mechanisms underlying these associations.

## Supplementary information

Supplementary Information

Supplementary Tables

## Data Availability

Data supporting the results of this study are available from official websites of China Health and Retirement Longitudinal Study (http://charls.pku.edu.cn) and Health and Retirement Study (https://hrs.isr.umich.edu/).
