# Supplementary material for: Association between depressive symptom and respiratory health in two prospective cohort studies
Source: NPJ Prim Care Respir Med. 2025 Dec 26;36:9. doi: 10.1038/s41533-025-00473-3 (PMC12855826; doi:10.1038/s41533-025-00473-3)
Supplement: Supplementary file 1 — Supplementary Information [file 41533_2025_473_MOESM1_ESM.docx]

**Supplemental Methods**

**Study designs of** **the CHARLS and HRS**

The China Health and Retirement Longitudinal Study (CHARLS) is a nationwide prospective cohort study in China. During the initial survey in 2011 (wave 1), 17,708 participants were enrolled from 28 provinces using a multistage probability sampling method. Although the primary inclusion criterion was age 45 years and above, a subset of individuals aged 40–44 also participated. Trained interviewers conducted structured face-to-face questionnaires to collect detailed information on sociodemographics, lifestyle behaviors, and health status. Anthropometric data were obtained from 13,978 participants, including measurements of height, weight, waist and hip circumferences, blood pressure, and handgrip strength. Follow-up evaluations occurred in 2013 (wave 2), 2015 (wave 3), and 2018 (wave 4), repeating both questionnaire interviews and anthropometric assessments. Additional blood samples were collected during the 2015 survey to reassess the same biochemical indicators.

The Health and Retirement Study (HRS) is a nationally representative, prospective cohort study in the United States. The initial wave in 1992 enrolled individuals aged 51–61 years, and was subsequently combined with the Asset and Health Dynamics of the Oldest Old Study, which recruited participants aged 70 years and above in 1993. To achieve full representation of the U.S. population aged 50 years and older, the study incorporated two additional cohorts in 1998, namely the Children of the Depression and the War Babies studies. Participants have been surveyed biennially since 1992, with interviews collecting detailed information on sociodemographic characteristics, lifestyle behaviors, and health status. Prior to 2004 (wave 7), interviews were primarily conducted via telephone, except for participants aged 80 and older who were offered in-person assessments. From 2006 onward (wave 8), half of the cohort underwent enhanced face-to-face interviews (EFTF), which included physical measurements such as height, weight, waist circumference, blood pressure, and grip strength, while the remaining participants continued with telephone interviews.

**Proportional hazards assumption**

In the analysis of baseline depressive symptoms, Kaplan–Meier curves in both the CHARLS and HRS cohorts were visually inspected and showed some crossing; however, all Schoenfeld residuals tests were nonsignificant (*P* > 0.05), indicating no statistical evidence of violation of the proportional hazards assumption. Similarly, in the analysis of depressive symptom trajectories, although slight crossing was observed among Kaplan–Meier curves for different trajectory groups, the Schoenfeld residuals tests remained nonsignificant (*P* > 0.05), suggesting that residuals were not significantly associated with time. Taken together, these results support the appropriateness of the proportional hazards assumption, indicating that the hazard ratio estimates from the Cox regression models can be interpreted with confidence.


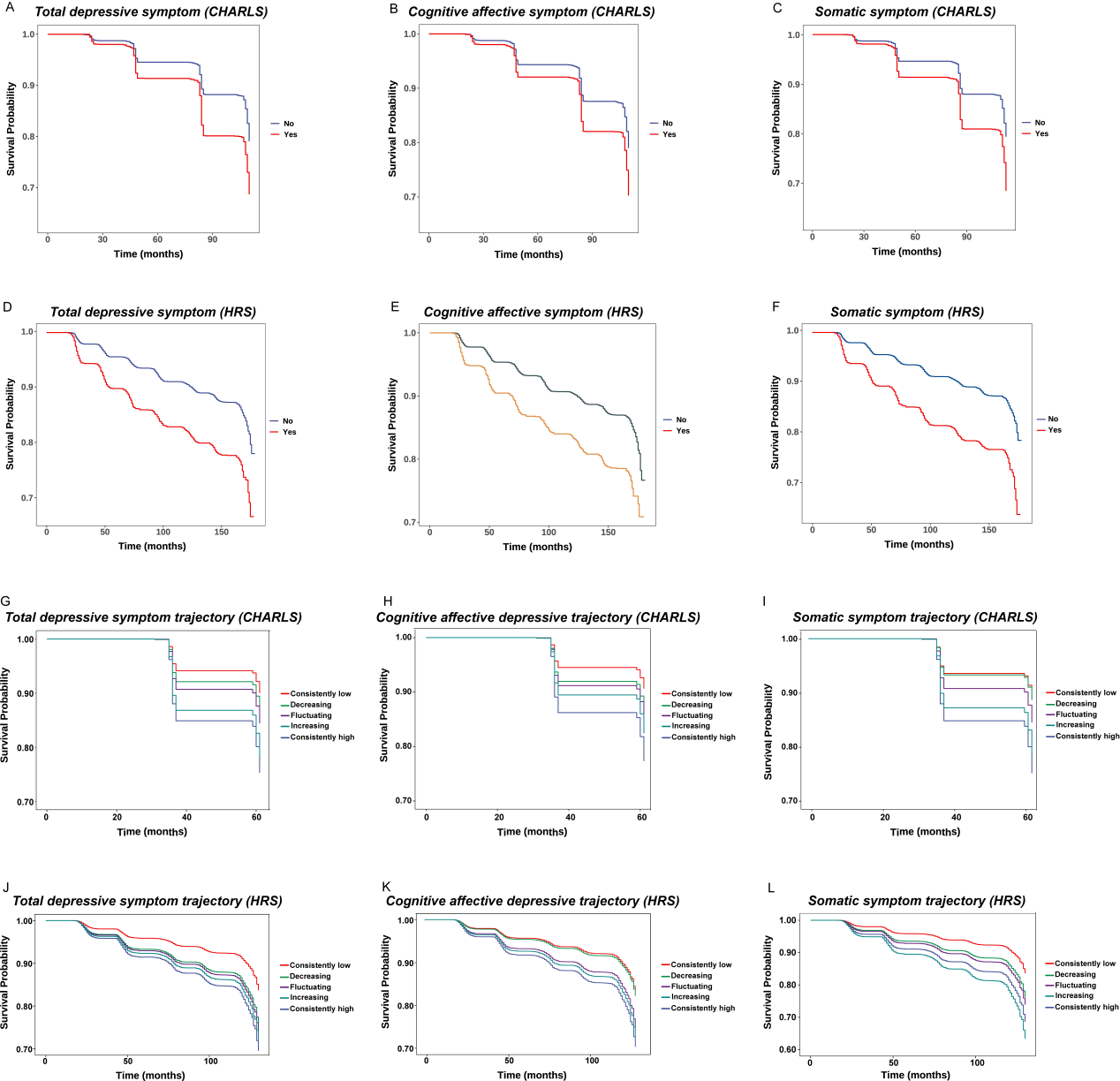


**Supplemental Figure 1.** Kaplan–Meier curves for the risk of CLDs according to depressive symptoms and their trajectories among middle-aged and older adults in the CHARLS and HRS cohorts. (Parallel group curves support the proportional hazards assumption)

Figure A, B, C, G, H and I are based on the CHARLS; Figure D, E, F, J, K and L are based on the HRS. Figure A, B, C, D, E and F show curves stratified by baseline depressive symptoms, including total depressive, cognitive-affective, and somatic symptoms. Figure G, H, I, J, K and L show curves stratified by the trajectories of total depressive, cognitive-affective, and somatic symptoms.

CHARLS, China Health and Retirement Longitudinal Study; HRS, Health and Retirement Study; CLDs, Chronic lung diseases.

**
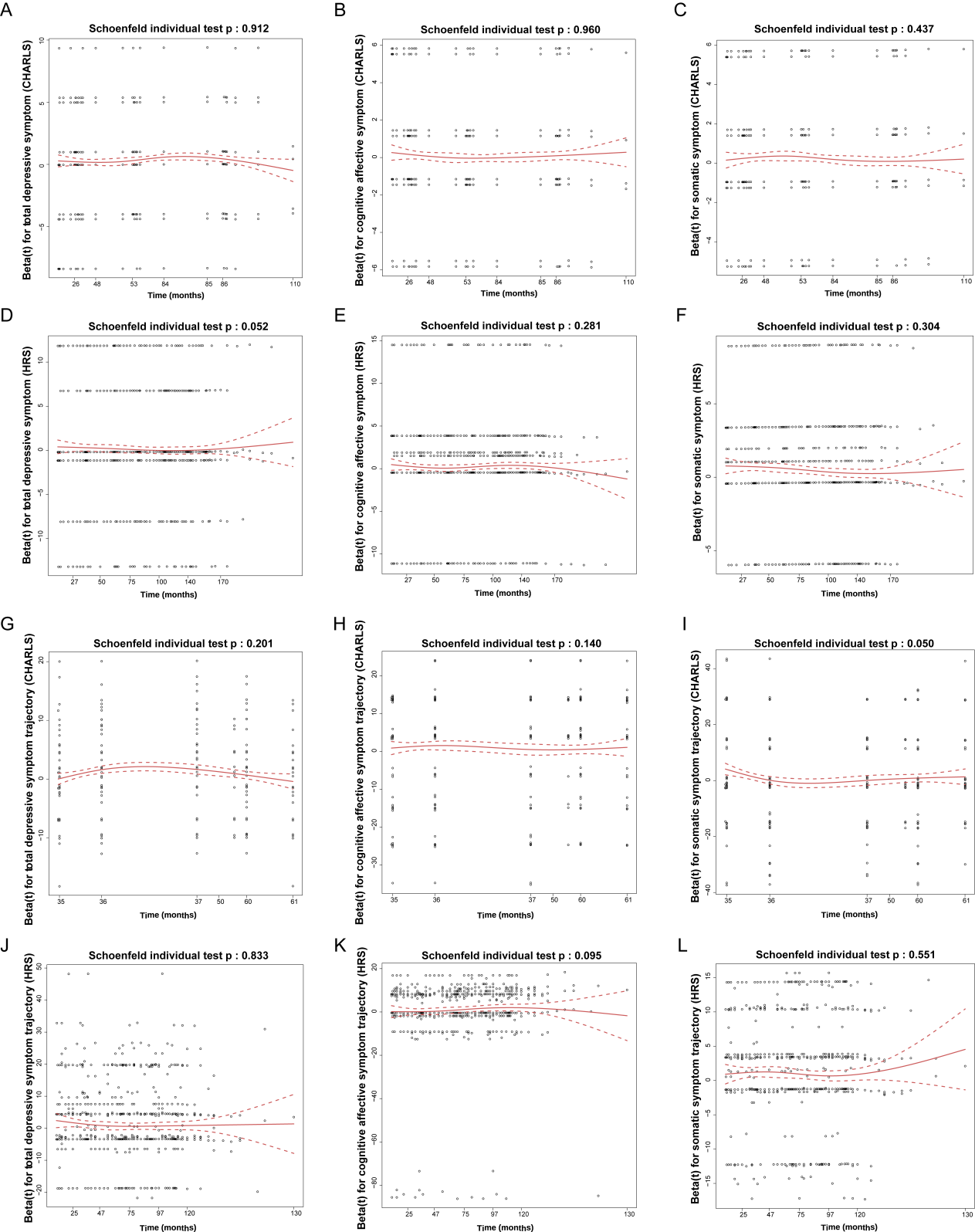
**

**Supplemental Figure 2.** Schoenfeld Residuals for the association of baseline depressive symptoms and their trajectories with CLDs in the CHARLS and HRS Cohorts. (Schoenfeld individual test *P* > 0.05 indicates no evidence to reject the proportional hazards assumption)

Figure A, B, C, G, H and I are based on the CHARLS; Figure D, E, F, J, K and L are based on the HRS.

Figure A, B, C, D, E and F show Schoenfeld residuals for baseline total depressive, cognitive-affective, and somatic symptoms. Figure G, H, I, J, K and L show Schoenfeld residuals for trajectories of total depressive, cognitive-affective, and somatic symptoms.

CHARLS, China Health and Retirement Longitudinal Study; HRS, Health and Retirement Study; CLDs, Chronic lung diseases.

**Covariate Selection**

Covariates were selected based on four main domains: Sociodemographic and socioeconomic factors (Age, Sex, Marital status, Education level), health behaviors (Smoking status, Drinking status, BMI), chronic diseases (Hypertension, Diabetes, Heart disease, Stroke), and sensory function (Hearing, Vision).

Age and sex are fundamental risk factors common to both chronic lung disease and depressive symptoms^[1, 2]^. Marital status and educational attainment serve as proxies for socioeconomic status, influencing access to healthcare, health literacy, and psychological health risk, which may also impact pulmonary health outcomes^[3, 4]^. Smoking is a major established environmental risk factor for chronic lung disease and has bidirectional associations with depression^[5, 6]^. Alcohol consumption and BMI are also associated with systemic inflammation and depressive symptoms^[7-9]^. Hypertension, diabetes, cardiovascular disease, and stroke may affect lung health through systemic inflammation and vascular dysfunction. These comorbidities are also well-recognized predictors of depression^[10, 11]^. Hearing and vision impairment are considered sensitive markers of systemic health decline and biological aging^[12, 13]^. They are associated with systemic inflammation, oxidative stress, and microvascular dysfunction, which may be related to chronic lung disease. Moreover, sensory impairments can lead to social isolation and functional limitations, which are strongly linked to depressive symptoms^[14, 15]^. Therefore, although hearing and vision may not be direct etiological factors for chronic lung disease, including them as potential confounders allows for more comprehensive adjustment of confounding bias between chronic lung disease and depressive symptoms.

**Reference**

1. Reddy KD, Oliver BGG: **Sexual dimorphism in chronic respiratory diseases.** *Cell Biosci* 2023, **13:**47.

2. Schuliga M, Read J, Knight DA: **Ageing mechanisms that contribute to tissue remodeling in lung disease.** *Ageing Res Rev* 2021, **70:**101405.

3. Wang Y, Liu M, Yang F, Chen H, Wang Y, Liu J: **The associations of socioeconomic status, social activities, and loneliness with depressive symptoms in adults aged 50 years and older across 24 countries: findings from five prospective cohort studies.** *Lancet Healthy Longev* 2024, **5:**100618.

4. Guo B, Wang Y, Pei L, Yu Y, Liu F, Zhang D, Wang X, Su Y, Zhang D, Zhang B, Guo H: **Determining the effects of socioeconomic and environmental determinants on chronic obstructive pulmonary disease (COPD) mortality using geographically and temporally weighted regression model across Xi'an during 2014-2016.** *Sci Total Environ* 2021, **756:**143869.

5. Chu WM, Nishita Y, Tange C, Zhang S, Furuya K, Shimokata H, Otsuka R, Lee MC, Arai H: **Association of second-hand smoke exposure combined with cigarette smoking and the development of depressive symptoms among middle-aged and older adults in Japan: 20-year population-based cohort study.** *Br J Psychiatry* 2025**:**1-8.

6. McRobbie H, Kwan B: **Tobacco use disorder and the lungs.** *Addiction* 2021, **116:**2559-2571.

7. Blüher M: **An overview of obesity-related complications: The epidemiological evidence linking body weight and other markers of obesity to adverse health outcomes.** *Diabetes Obes Metab* 2025, **27 Suppl 2:**3-19.

8. Gerardo G, Peterson N, Goodpaster K, Heinberg L: **Depression and Obesity.** *Curr Obes Rep* 2025, **14:**5.

9. Pommerolle L, Arif M, Behee M, Appolonia CN, Basu A, Wolf KM, Zawatsky CN, Johnson N, Rivellini O, Park JK, Cinar R: **Chronic Alcohol Intake Compromises Lung Immunity by Altering Immunometabolism in Humans and Mouse Models.** *Am J Respir Cell Mol Biol* 2024, **71:**559-576.

10. Scott AJ, Correa AB, Bisby MA, Dear BF: **Depression and Anxiety Trajectories in Chronic Disease: A Systematic Review and Meta-Analysis.** *Psychother Psychosom* 2023, **92:**227-242.

11. Li S, Jia Z, Zhang Z, Li Y, Ding Y, Qin Z, Guo S: **Effect of gender on the association between cumulative cardiovascular risk factors and depression: results from the US National Health and Nutrition Examination Survey.** *Gen Psychiatr* 2023, **36:**e101063.

12. Aliyeva A, Sari E: **Evaluation of autoimmune and inflammatory markers in bilateral sudden hearing loss.** *Eur Arch Otorhinolaryngol* 2025, **282:**4637-4644.

13. Vohra V, Simonsick EM, Kamath V, Bandeen-Roche K, Agrawal Y, Rowan NR: **Physical Function Trajectories and Mortality in Older Adults With Multisensory Impairment.** *JAMA Otolaryngol Head Neck Surg* 2024, **150:**217-225.

14. Zhang Y, He X, Liu Y, Tian C, Yang H, Zhang L: **The association between hearing loss and depression in the China health and retirement longitudinal study.** *Sci Rep* 2025, **15:**20537.

15. Wang EB, Garcia Morales EE, Gross AL, Lin FR, Reed NS, Deal JA: **Residential Differences and Depression Among Older Adults With Dual Sensory Loss.** *JAMA Otolaryngol Head Neck Surg* 2025, **151:**202-210.
